# Evaluation of the Cost of Survivorship Care After Allogeneic Hematopoeitic Stem Cell Transplantation–An Analysis of 2 German Transplantation Centers

**DOI:** 10.3389/fpubh.2020.572470

**Published:** 2020-09-25

**Authors:** Daniel Wolff, Jelena Bardak, Matthias Edinger, Ursula Klinger-Schindler, Ernst Holler, Anita Lawitschka, Helene Schoemans, Wolfgang Herr, Nikolaus Kröger, Francis Ayuk Ayuketang

**Affiliations:** ^1^Department of Internal Medicine III, University Hospital Regensburg, Regensburg, Germany; ^2^Management Consulting for Clinic and Medizinisches Versorgungszentrum (MVZ), Berlin, Germany; ^3^St. Anna Children's Hospital, Stem Cell Transplantation (SCT)-Outpatient and Aftercare Clinic, Medical University Vienna and Children's Cancer Research Institute, Vienna, Austria; ^4^Department of Hematology, University Hospitals Leuven, KU Leuven, Leuven, Belgium; ^5^Department of Stem Cell Transplantation, University Hospital Hamburg-Eppendorf, Hamburg, Germany

**Keywords:** GvHD, Graft vs. Host disease, haematopoietic stem cell transplant, survivorship care model, costs, re-imbursement

## Abstract

The aim of the presented study was to analyze the care expenditure for outpatients after allogeneic hematopoietic stem cell transplantation (alloHSCT) done in accordance with the national, European guidelines and the German Social Law. We performed an analysis of the National and European survivorship care guidelines and in parallel recorded the time expenditure and staff costs separated according to different occupational groups involved in outpatient care at two German transplantation centers [University Hospital Regensburg (UKR) and University Hospital Hamburg-Eppendorf (UKE)]. In addition, we performed a comparison of real costs vs. reimbursed costs according to the standard rating benchmark catalog (EBM), which was supplemented by a survey of German transplantation centers. The results showed that the staff costs are only covered by the EBM for patients without complications during long-term follow-up care—notably, this accounts for 15% of alloHSCT patients. Staff costs for patients requiring treatment of graft-vs.-host disease or relapse of the malignant underlying malignancy exceed to the factor 6.5 (UKR) to 12 (UKE) of the EBM revenue, caused both by the increased duration and frequency of the outpatient visits. As a result of the survey at German transplant centers, 15 out of 18 responding centers reported a lack of cost coverage for follow-up care. Two/15 centers reported that survivorship care is limited to a restricted time, independent of patient's needs, due to a lack of cost reimbursement. The results show that alloHSCT survivorship care of patients requires significant staff resources, which are not covered by the current version of the German EBM catalog. New approaches to finance labor intensive after care of transplant patients are required.

## Introduction

The lifelong survivorship care of patients after allogeneic haematopoietic stem cell transplantation (alloHSCT) is becoming increasingly important due to the rising number of long-term survivors. AlloHSCT survivorship care requires a special expertise due to transplantation-specific long-term complications such as Graft-vs.-Host disease (GvHD) (1) as outlined by the German social law, the JACIE guidelines (JACIE: Joined Accreditation Committee of the International Society for Stem Cell Transplantation (ISCT) for the accreditation of transplantation centers), and national and European guidelines (2, 3). In particular, an intensified survivorship care that is individually adapted to the patient is indispensable in order to maintain the patients' quality of life despite multimorbidity, also due to aging transplant patients with increased morbidity as advocated by both the Institute of Medicine and the NIH Late effects initiative (4, 5). Moreover, an multicenter analysis performed at transplant centers in the United States revealed an improved long-term survival of transplant survivors when dedicated survivorship care was available (6). However, the resulting increase in the personnel costs, mainly within an outpatient setting, requires adequate re-compensation. In Germany, financial coverage of outpatient services is currently ensured by the standard rating benchmark catalog (EBM). While the EBM was designed for the remuneration of specialist outpatient services and is based on a mixed calculation and consecutive quarterly flat rates, it has so far not been adapted to the distinct circumstances of specialized university hospital outpatient clinics, which potentially leads to a significant underfinancing and consecutive restriction of services in the aftercare of transplant patients. In the present research project we analyzed the institutional care expenditure required to provide survivorship care according to the German and European guidelines and compared the results to the current version of the EBM catalog and developed subsequent suggestions to resolve the identified discrepancies.

## Methods

We performed a comparative analysis of the time effort of the survivorship care after alloHSCT (2, 3) at two outpatient departments of the University Hospital Regensburg (UKR) and the University Hospital Hamburg—Eppendorf (UKE) in order to account for center-specific differences. At the time of the analysis, both centers had a current JACIE certification and thus carried out survivorship care in accordance with German and European alloHSCT guidelines. During each 2-weeks period, data of 100 patients (50 patients per hospital) were collected during regular outpatient visits. The data collection was carried out by a direct work time recording of the three different occupational groups directly involved in patients individual care such as physicians, physician assistants and nurses. Additionally, a survey on the organizational structure of alloHSCT survivorship care was completed.

The time of direct patient contact during individual appointment was recorded separately for the three predefined occupational groups using a stopwatch. In addition, the duration of the preparation and post-processing of the medical records and findings of each individual patient was recorded. With these time measurements data sets for individual patient visits were created. The mean values were calculated from the individual data sets and used for the calculation. The required clinical data were taken from the medical records of the hospital information system. In order to calculate the total time per 3-months interval, the mean time required per patient visit was multiplied by the number of visits. The decision to analyze only 50 patients per center was based on the results of a pilot phase, showing that the time requirements for individual patient visits did not vary significantly, and the number of visits per 3 months interval per patient group (defined below) did not vary significantly too.

In order to reflect the high heterogeneity of care expenditure in follow-up (e.g., outpatient visits of patients with active GvHD requiring visits twice a week with a direct patient contact of 30 min each vs. outpatient visits without transplant-associated complications requiring visits every 3 months with a direct patient contact of 15 min) six different reference categories were defined (Table 1). Active GvHD was defined by GvHD requiring immunosuppressive treatment. Patients having concomitant GvHD and relapse were excluded from the analysis.

**Table 1:** Reference categories of patient groups.

**Reference categories**	**% of pts. UKR** ***n* = 50**	**% of pts. UKE** ***n* = 50**
**Patients with active acute or chronic GvHD (GvHD):** patients treated for acute or chronic GvHD	52	34
**Patient with relapse of the underlying malignancy (R):** therapy of a relapse of the underlying malignancy after alloHSCT	10	18
**Patient without GvHD but complications (C):** infections, secondary malignancies, multimorbidity in the absence of GvHD	8	12
**Early standard survivorship care (Se):** first outpatient month post alloHSCT	2	4
**Intermediate standard survivorship care (Si):** 3–12 months post alloHSCT	8	22
**Long-term standard survivorship care (Sl):** >12 months post alloHSCT	20	10

Patients with acute or chronic GvHD, patients treated for a relapse of the underlying disease and patients with other complications (e.g., infections, or secondary malignancies in the absence of GvHD) were classified. Patients in the “standard survivorship care” category were divided into “early,” “intermediate,” and “long-term” according the time point post alloHSCT (one, two-12 and ≥12 months post hospital discharge). All adult patients with hematological diseases as indication for alloHSCT were included in the data collection. Of note, only patients with public health insurance were included, as privately insured patients are not billed according to EBM catalog but account for only 10% of transplanted patients.

The remuneration for patient care was determined according to the patients' age and the associated EBM number derived from the EBM catalog. Only care costs incurred by the transplant outpatient department itself were taken into account. An analysis of costs derived from laboratory or radiological diagnostics was not included, as these are represented as standardized individual services in the EBM catalog and are neither alloHSCT—specific nor remunerated by a flat rate per quarter independent of the actual application. Furthermore, the costs of the basic equipment were not included.

The medical staff costs were calculated by offsetting the results of the medium time expenditure per patient category against the minute wage for the occupational group derived from the StepStone salary report 2016 (www.stepstone.de). The minute wage was 0.56€^*^ for doctors (experienced fellow), 0.21€^**^ for medical assistants, and 0.26€^***^ for nursing staff.

In addition, we surveyed German transplant centers on the organizational structure of alloHSCT aftercare. A paper-based questionnaire was sent by e-mail to 56 German transplant centers, 18 of which (32%) replied. The questionnaire contained 14 questions (combination of multiple-choice format and the possibility to provide answers in an unstructured format) focusing on billing models including problems of cost recovery, applications for cost coverage for the use of off-label drugs and coverage of transport-costs to the transplant center, the number of patients treated per quarter, and structures of survivorship care including possible time limitation.

## Results

The total time expenditure per 3 months at the UKR for physicians was a medium of 220 min (8.9 visits a 24.77 min) for GvHD patients, 349.74 min (17.4 visits a 20.1 min) for patients treated for relapse of the underlying malignancy, 147.88 min (6.5 visits a 22.75 min) for patients with infectious complications or multimorbidity independent of GvHD, 282 min (12 visits a 23.5 min) for early standard survivorship care, 200 min (10 visits a 20 min) for intermediate standard and 18.7 min (1 visit) for long-term standard survivorship care patients.

Medical assistants (MA) spend an average of 308.91 min per 3 months for GvHD patients, 523.74 min for patients with relapse, 212.88 min for patients with complications, 402 min for standard early survivorship care patients, 300 min for intermediate standard survivorship care and 28.7 min for long-term standard survivorship care patients.

**Figure 1:**
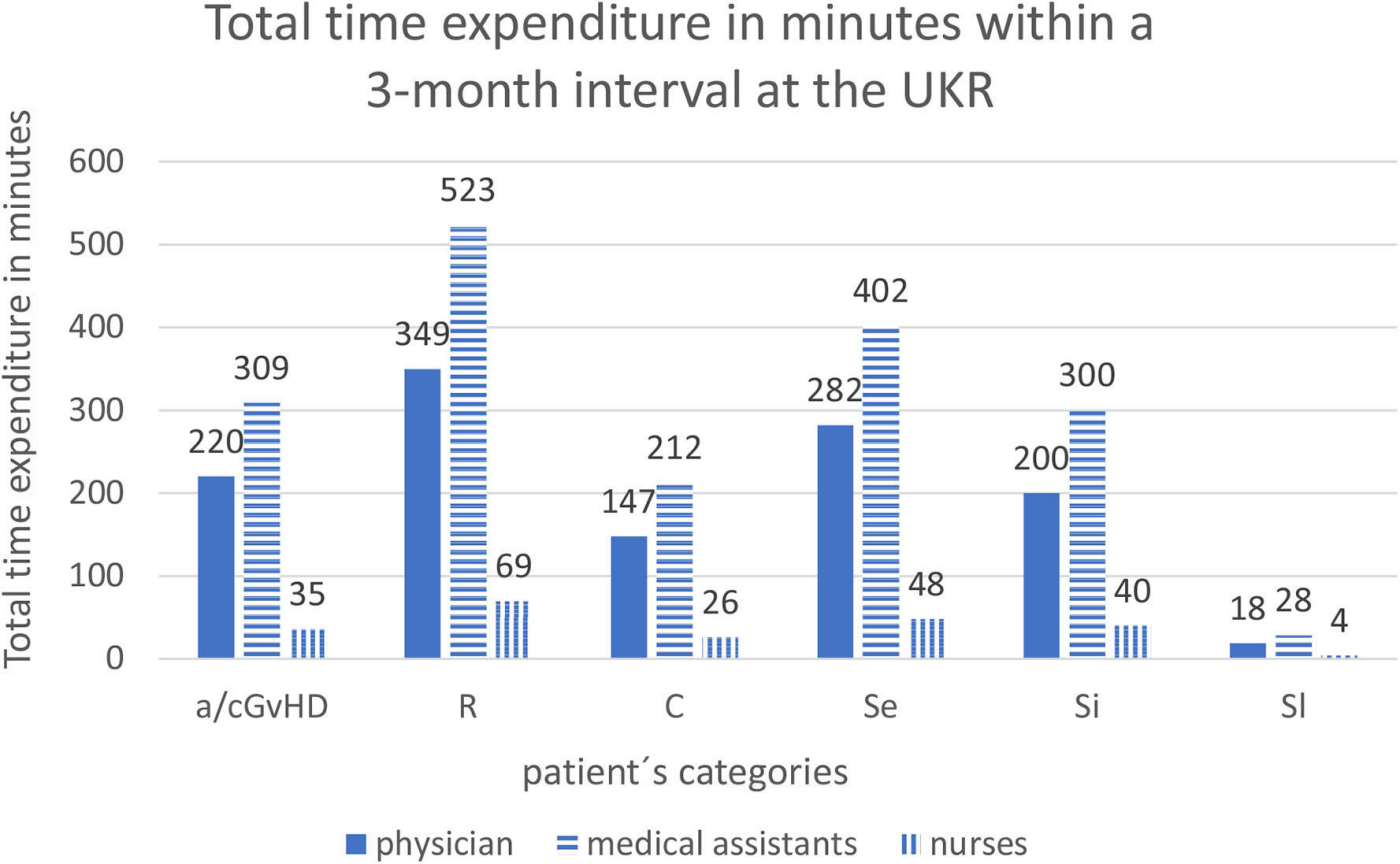
Total time expenditure in minutes within a 3-months interval at the UKR. Abbreviations of patient's categories are depicted in Table 1.

For nurses (*N*), the required time required for care within a 3 months interval of GvHD patients was 35.54 min, 69.6 min for relapsed patients, 26 min for patients treated for complications, 48 min for standard early follow-up, 40 min for standard intermediate follow-up, and 4 min for standard long-term follow-up (Figure 1).

At the UKE, the total time expenditure for physicians during 3 months was a medium of 256.8 min (12.76 visits a 20.12 min) for GvHD patients, 227.37 min (11 visits a 20.67 min) for relapsed patients, 199.5 min (10.5 visits a 19 min) for patients with complications, 258 min (12 visits a 21.5 min) for early standard survivorship care, 118.6 min (6.1 visits a 19.45 min) for intermediate standard survivorship care and 19.6 min (1 visit) for long-term standard survivorship care.

**Figure 2:**
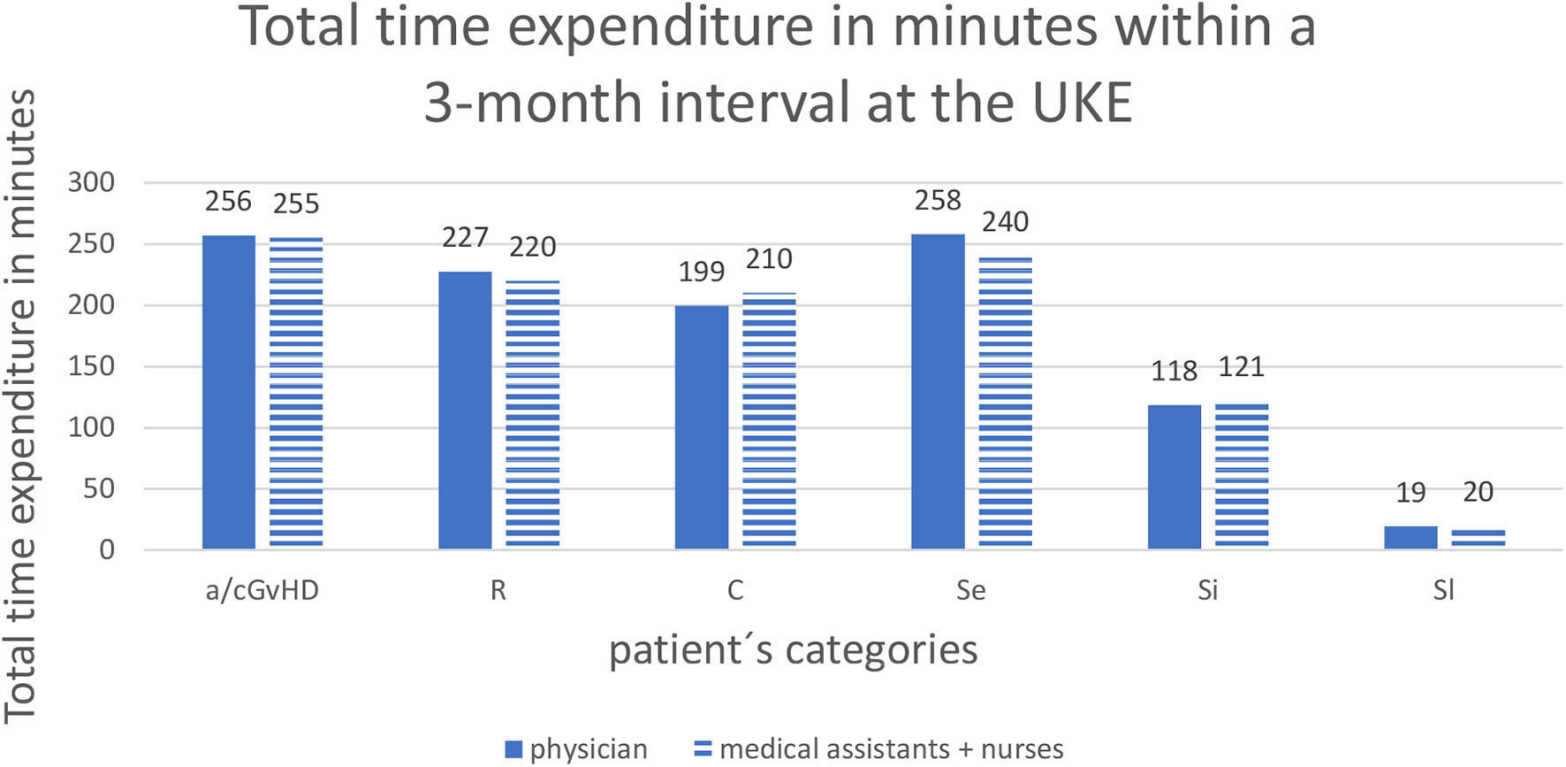
Total time expenditure in minutes within a 3-months interval at the UKE. Abbreviations of patient's categories are depicted in Table 1.

Medical assistants spend during a 3-month interval a medium of 255.29 min for GvHD patients, 220 min for relapsed patients, 210 min for patients with complications, 240 min for early standard follow-up, 121.82 min for intermediate standard follow-up, and 20 min for long-term standard follow-up (Figure 2).

**Figure 3:**
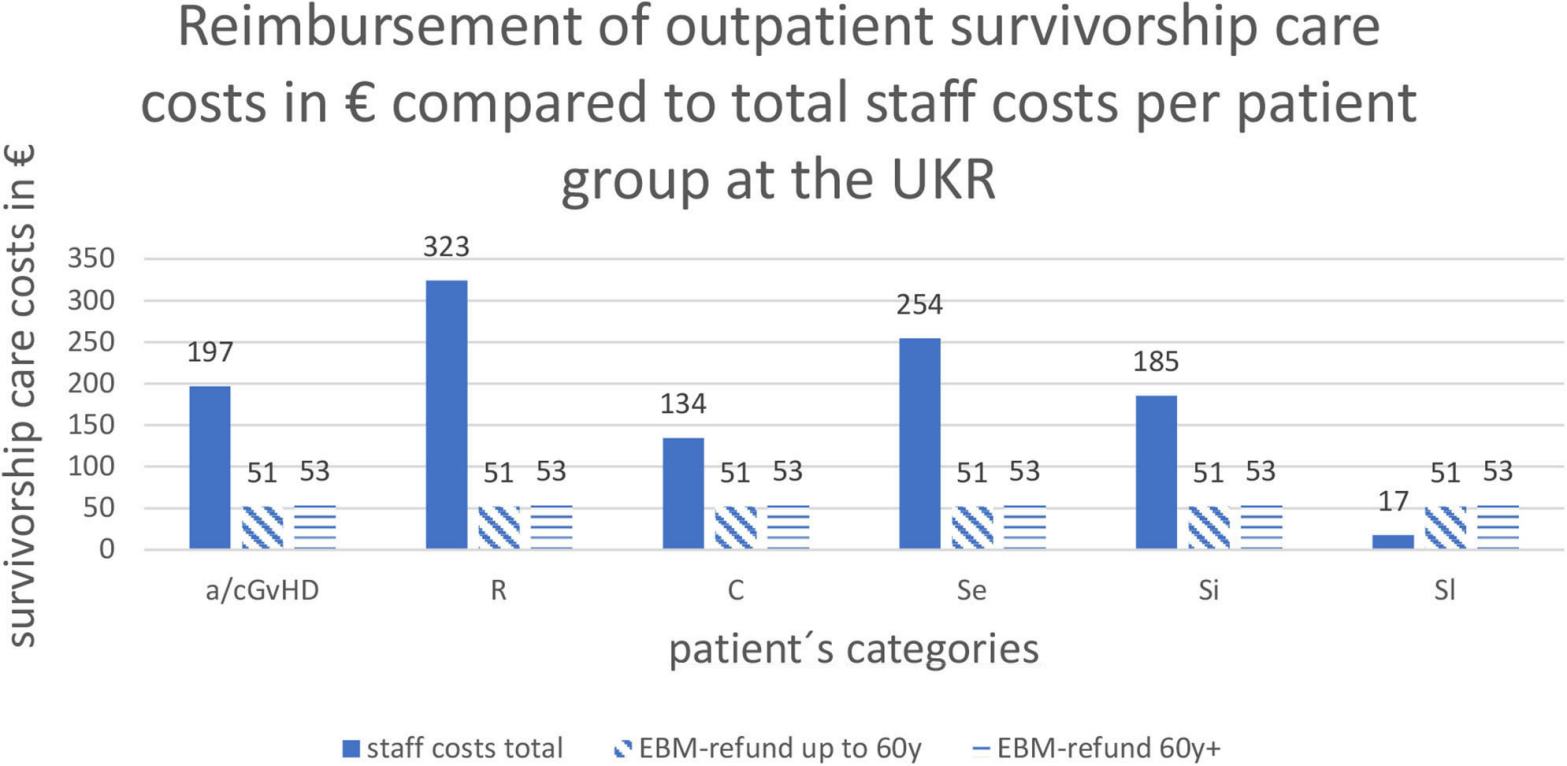
Reimbursement of outpatient survivorship care costs in € compared to total staff costs per patient group at the UKR: Abbreviations of patient's categories are depicted in Table 1.

The alloHSCT outpatient clinic at the UKR, which is reimbursed according to federal regulations for specialized care at university hospitals (§116b) directly from the insurance companies, received a flat rate of 51.86€ (<60 years) or 53.33€ (> 60 years) per 3-months interval irrespective of the number and length of individual visits. The lump sum consisted of the basic rate for patients with a public insurance (No. 13491 > 60 years, No. 13492 > 60 years) and an extra fee for patients after alloHSCT (No. 13501 equal of 19.93€) (Figure 3).

**Figure 4:**
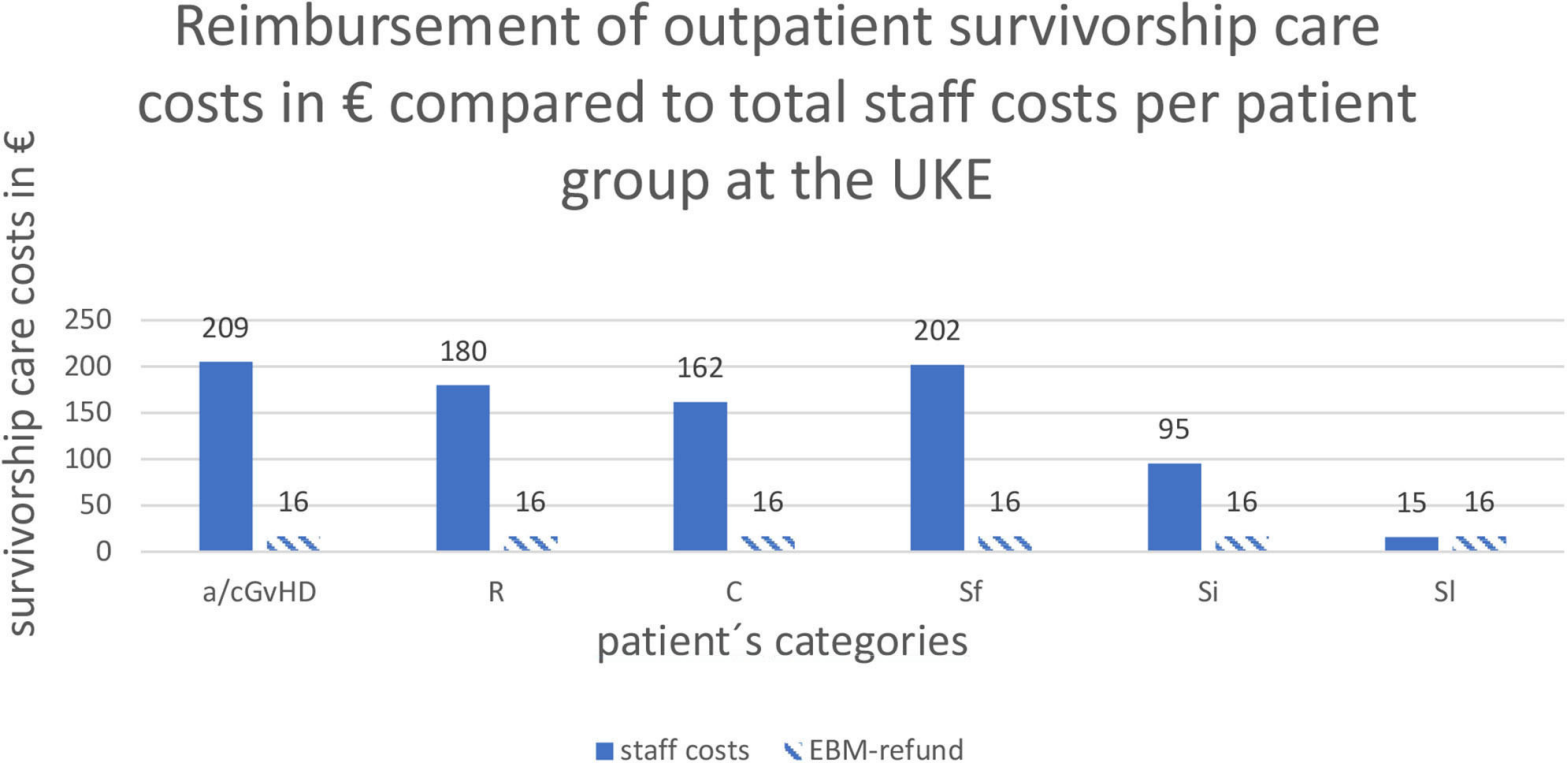
Reimbursement of outpatient survivorship care costs in € compared to total staff costs per patient group at the UKE: Abbreviations of patient's categories are depicted in Table 1.

The alloHSCT outpatient clinic at the UKE, which is reimbursed according to the rules of standard outpatient care in internal medicine for authorized doctors, institutes and hospitals from the Association of Public Health Insurance Physicians (“Kassenärztliche Vereinigung”) was only able to bill 16.59€ (No. 01321) for the care expenditure per 3-months interval per patient regardless of the frequency of visits (Figure 4).

## Results of the Survey

**Figure 5:**
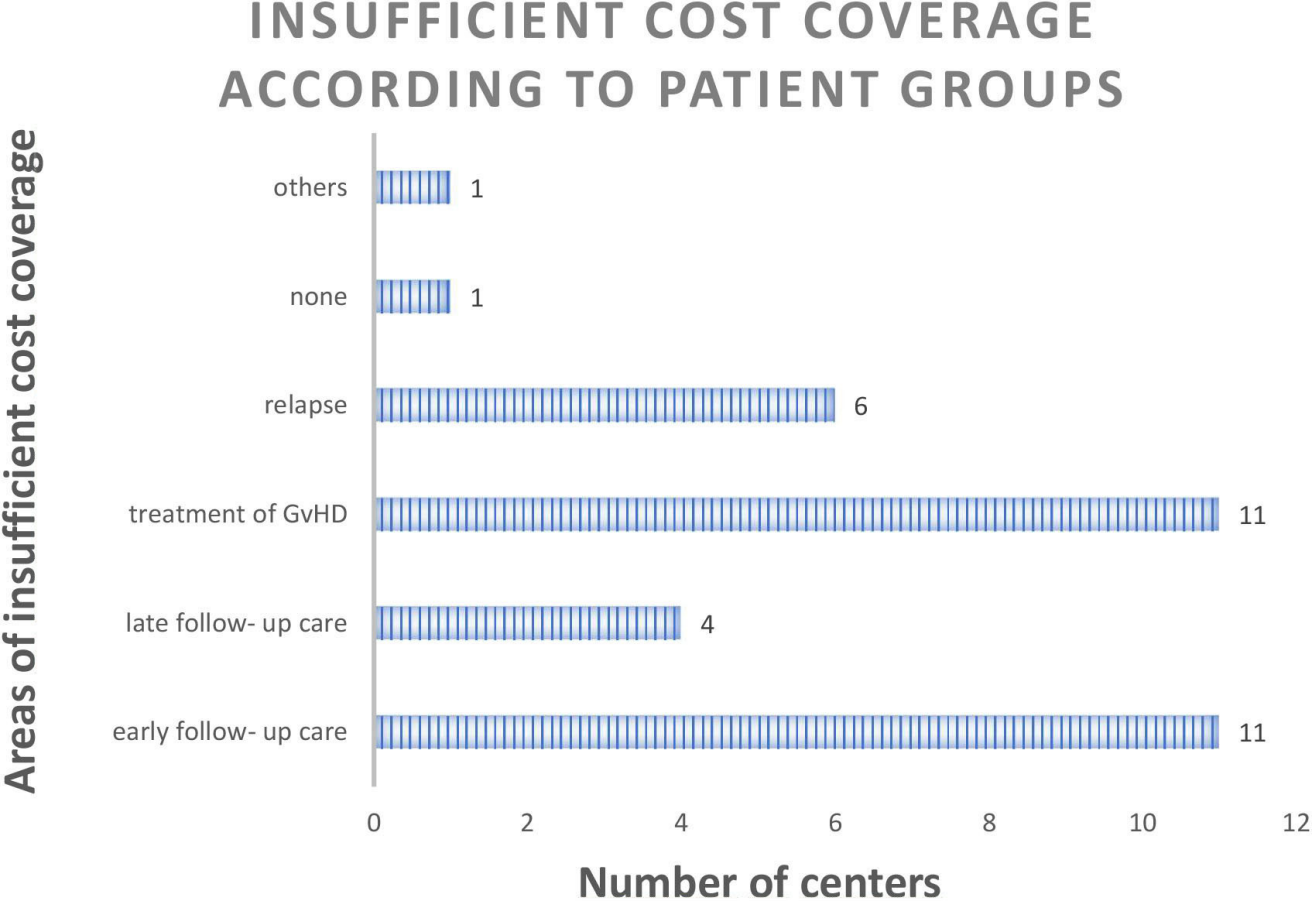
Number of centers (total number of replying centers *n* = 18) with insufficient cost coverage according to patient groups *(multiple answers possible)*.

The evaluation of the survey reported a high rate (15 of 18 responding centers taking care for a survivorship care of 117 patients per 3-months interval) of billing problems and 14 centers (78%) reported an insufficient cost recovery of staff costs. Specifically, 61% of the centers stated that the reimbursement of early and intermediate survivorship care after transplantation is not covered by the public health insurance reimbursement. The same applies to coverage of treatment of GvHD patients. In addition, 33% of centers complained on insufficient cost recovery for treatment of patients with relapse, or late follow-up (22%). Another area of insufficient coverage was treatment of complex multimorbid patients with numerous necessary expensive diagnostic examinations or severe infections (see Figure 5).

An additional reported issue independent of lack of coverage of staff costs was the absence of an outpatient reimbursement for extracorporeal photopheresis (ECP) and donor lymphocytes infusions (DLI), difficulties with billing of laboratory costs and the non-existent reimbursement of vaccination after alloHSCT. Moreover, it was reported, that survivorship care for patients receiving an alloHSCT for non-malignant disease can't be reimbursed according to federal regulations for specialized care at university hospitals (§116b). This applies in particularly to children's hospitals, since non-malignant diseases requiring transplantation, such as haemoglobinopathies, metabolic defects, and immunodeficiencies, account for about 30% of all pediatric alloHSCT.

**Figure 6:**
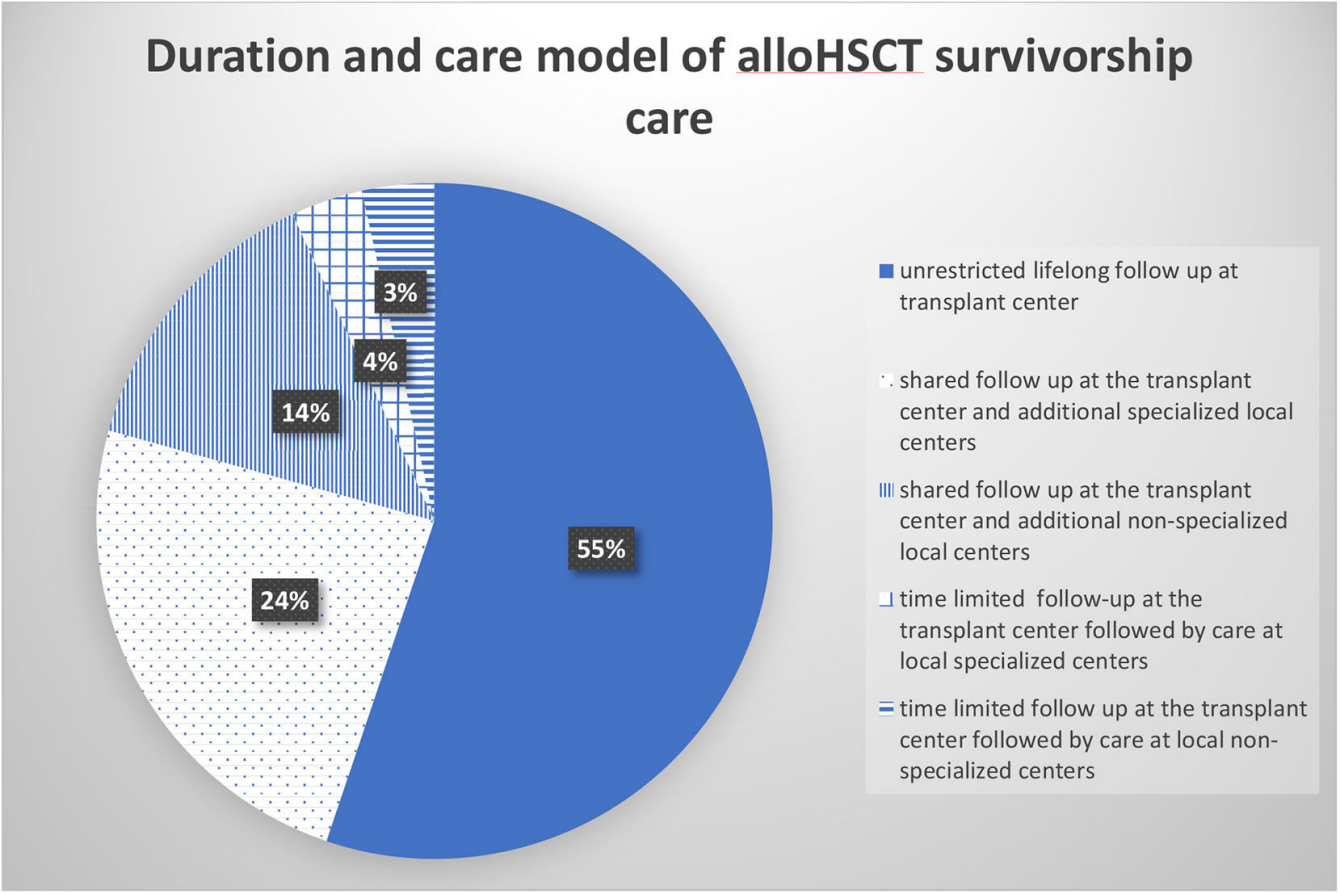
Duration and care model of alloHSCT survivorship care (percentage of total number of responding centers *n* = 18).

Sixteen of the 18 replying centers provided a lifelong survivorship care with 24% of centers supplementing the survivorship care at specialized outpatient facilities while 14% of the replying centers involved local survivorship care lacking alloHSCT expertise. Two of the 18 centers provided time limited survivorship care only (see Figure 6).

The main billing models applied for re-compensation were university outpatient contracts, individual contracts with the Association of Public Health Insurance Physicians, or reimbursement according to federal regulations for specialized care at university hospitals (§116b). Of note, all three re-compensation models apply a fixed flat rate independent of disease severity, number of visits and staff costs per 3-months interval.

Of interest, 10 centers used multiple re-compensation systems at the same time. Additional re-compensation systems applied were the use of day clinics or billing to Association of Public Health Insurance Physicians within a Medical Care Center (MVZ).

The billing rates were calculated using the standard rating benchmark catalog (EBM) at 15 of the 18 centers (83%). Six centers also applied the diagnosis related groups (DRG) catalog (originally designed for inpatient re-compensation) to cover costs of ECP, DLI and blood transfusions, which are not covered within the EBM catalog.

## Discussion

The results show that the survivorship care of patients following alloHSCT according to the German federal law and National and European guidelines requires a high staff expenditure. However, the revenues generated by the standard rating benchmark catalog are not cost covering for the vast majority of patients. This is mainly due to the high frequency, while prolonged duration of individual visits required by the clinical status of the patients contribute. In contrast, the current version of the standard rating benchmark catalog (EBM) covers only 1−2 visits applying a flat rate for a 3-months interval independent of actual time requirements. This leads to a discrepancy between staff costs and revenues of 3−6 times (UKR) and up to 12 times (UKE) depending on the patient group and institution. The only patient group with complete cost coverage were long-term standard survivorship care patients, since for the latter only one contact per 3-months interval or less was required. However, this applies only to 15% of the alloHSCT patient population.

Other revenues to be generated for covering outpatient visits relate in particular to diagnostic procedures and therapies, which are covered by the standard rating benchmark catalog. However, these revenues just cover costs of the diagnostic procedures and do not cover staff costs of the transplant outpatient department. The problem of inadequate remuneration can be historically explained by the fact that the standard rating benchmark catalog (EBM) was not developed for the survivorship care of transplant patients and therefore no cost calculation was made for the care of the latter population.

The possibility to improve the efficiency of outpatient clinics by optimizing time schedules will not be able to resolve the situation, as the comparative analysis of paper-based documentation at the UKR (which was associated with higher staff costs due to a higher time expenditure) had only insignificant higher staff costs compared to the completely digitalized and optimized system used at the UKE.

One possible solution would be the implementation of additional standard rating benchmark catalog procedures covering costs of the individual patient visits of during alloHSCT survivorship care. This addition would not be required for survivorship care of long-term patients, since for the latter population the reimbursement is already cost covering. Alternatively, staggered flat rates could be implemented covering the average cost of alloHSCT survivorship care for the different patient categories mentioned above.

The new outpatient specialist medical care constructs (ASV) according to the social law (§116b SGB V) seems to be the most promising construct, since it permits interdisciplinary and cross-sector care and coordination services are also remunerated (7). In addition, the care structures required by ASV contracts are relatively congruent with the care structures required by the national and international guidelines including JACIE certification. Furthermore, according to the ASV guidelines, transplant patients represent a group with rare diseases (acute and chronic GVHD) and special needs and can be defined by the transplantation ICD-10 number. Furthermore, the ASV potentially offers the option of mapping typical services (ECP, DLI) on an outpatient basis. So far, however, there is no ASV specification implemented for patients after alloHSCT. An additional measure to reduce staff costs may be the implementation of eHealth strategies which would permit remote monitoring of patients and thereby may reduce the number of visits but requires re-compensation as well.

The results of the survey show that the inadequate renumeration of survivorship care has direct consequences for the patients concerned. Although only 1/3 of the German centers took part in the survey, the vast majority of centers reported a lack of cost recovery for survivorship care associated with time limitations and therefore, possibly serious consequences for patients. These real-life approaches clearly contradict the requirements of the federal social law (which requires survivorship care at the alloHSCT center) and National and European guidelines (3, 8).

The analyses demonstrate that the current form of standard rating benchmark catalog (EBM) based billing does not meet the requirements of guideline-oriented patient alloHSCT survivorship care, and that the lack of cost recovery impacts the quality of survivorship care. An adaptation of the catalog and the creation of new remuneration structures (e.g., ASV) are therefore urgently needed to ensure the coverage of costs for survivorship care of alloHSCT survivors.

## Data Availability Statement

The raw data supporting the conclusions of this article will be made available by the authors, without undue reservation.

## Author's Note

The analysis was performed in the framework of the COST project CA17138 cGvHD Eurograft.

## Author Contributions

DW: designed the study, wrote the manuscript, and supervised the analysis. JB: performed the study and wrote the draft of the manuscript. ME: contributed to the manuscript and supervised the study. UK-S: contributed to the results section and discussion (billing models). EH: supervised the study. AL: corrected the manuscript and added to the discussion. HS: added to the discussion, corrected the manuscript, and discussed the conclusions. WH: supervised the study. NK: supervised the study at UKE. FA: designed the study at UKE and corrected the manuscript. All authors contributed to the article and approved the submitted version.

## Conflict of Interest

The authors declare that the research was conducted in the absence of any commercial or financial relationships that could be construed as a potential conflict of interest.
